# Healthcare AI, explainability, and the human-machine relationship: a (not so) novel practical challenge

**DOI:** 10.3389/fmed.2025.1545409

**Published:** 2025-02-28

**Authors:** Claudia Giorgetti, Giuseppe Contissa, Giuseppe Basile

**Affiliations:** ^1^CIRSFID, Department of Legal Studies, University of Bologna, Bologna, Italy; ^2^Department of Biomedical Science and Public Health, University “Politecnica delle Marche” of Ancona, Ancona, Italy

**Keywords:** explainability, AI, healthcare, human-machine relationship, informed choice, evidence-based medicine, liability, XAI

## Abstract

This paper focuses on the lack of explainability that afflicts machine-learning-based AI systems applied in the field of healthcare. After a brief introduction to the topic, from both a technical and legal point of view, this work aims to assess the main consequences that the lack of explainability has on the human-machine relationship in clinical care, through a practical perspective. It then questions whether explainability is truly an objective worth seeking and, if so, to what extent, taking into account the current possible solutions.

## 1 Introduction

Artificial Intelligence (AI) applications in the field of healthcare can provide numerous benefits that are destined to significantly impact all medical professions in the very near future. Growing investments in the field, as financed by Big Tech companies (1), have resulted in increasing enthusiasm around Clinical Decision Support Systems (CDSSs) (2), and, more specifically, Diagnostic Decision Support Systems (DDSSs) (3), which nowadays are mostly based on machine learning^1^ (ML) (4). While many experts push for widespread adoption of this technology, it must not be forgotten that AI is an added element of complexity. As such, while on one hand, it aims to simplify processes, on the other it can present new issues for the medical domain that must be evaluated and addressed.

## 2 Explainability (or a lack thereof)

AI systems based on ML methods present significant issues concerning explainability. These systems have, in fact, often been described as “black boxes”, since they are unable to explain how or why they were able to reach a certain conclusion, i.e., to produce a certain output (5). Upon completion of the system’s training, the resulting algorithm is unknown, thus making it extremely difficult to fully understand and justify the machine’s outcomes (6).

Explainability is defined by the High-Level Expert Group (HLEG) Ethics Guidelines for Trustworthy AI (7) as “the ability to explain both the technical processes of an AI system and the related human decisions” (7). These Guidelines also mention a different term, explicability, among the principles of AI. According to Floridi et al. (8) it has to be understood as a broader concept, often used interchangeably with the term transparency, which indicates a combination of both intelligibility and accountability (8). It is important to point out that a system being transparent is not the same as it being explainable. As some authors have noted, transparency should be intended as a passive characteristic of the system, while explainability requires an active effort, from the system, to make itself understandable (9). In this sense, ensuring transparency does not necessarily guarantee explainability and vice-versa (10).

## 3 The issues caused, their origins, and their presumed novelty

The reasons why explainability assumes particular relevancy in the healthcare field are numerous, ranging from the identification of biases to the provision of new insights for research (11). In this work, though, the analysis will be limited to explainability’s significance in the interaction between humans and machines in the context of clinical care and from a concrete point of view.

First of all, it is essential to keep in mind that both the healthcare system in general and AI applications in healthcare are socio-technical systems (STSs), as such requiring cooperation between humans and machines to work properly (12). Some degree of human-machine interaction (HMI) is bound to always take place in light of the healthcare system’s overall characteristics. When it comes to singular tasks, though, it is important to note that this intercommunication could:

• Be imposed by ethical and legislative choices [e.g., the human oversight principle, Art. 14 of the Artificial Intelligence Act (AI Act)] (13).
• Be required by the concrete nature of the assignment at hand. There are, for example, some crucial inputs that the machine might be unable to pick up on (e.g., certain smells, certain skin textures), as well as some tasks that are based on interacting physically with the patient (e.g., using a stethoscope to gain information about respiratory diseases), that require the presence of a human.

In these situations that lack full automation, the physicians interacting with a DDSS might, for example, be asked to choose a therapeutic option among the ones proposed by the system or to come up with their own solution upon reflection on the options presented. Because of the highlighted difficulties, a “give or take” scenario could present itself. Since physicians are unable to fully understand the reasoning behind the machine’s output (and consequently the output’s meaning and weight), they might be essentially forced to choose between either fully relying on or completely disregarding the conclusion of the AI. The lack of explainability can, in fact, contribute to either Decision-Automation bias or Automation-Distrust bias (14).^2^ These so-called “intermediate levels of automation”, tend to present the most relevant difficulties precisely because of how critical this trade-off moment is (15). It would seem unfair to realistically expect the human to fully evaluate an output that he does not intimately understand, in what is usually also a very short amount of time. The risk, of course, is losing either the concrete impact of human contribution or the benefits of the AI system’s capabilities. Non-AI-powered technologies are certainly relied on by physicians, but they (usually) have been validated through years of research, have received widespread approval by the scientific community at large, and lack the unique challenges of AI (especially the unpredictability of AI systems)^3^. In this sense, the trust that healthcare professionals have in AI technology is significantly limited in comparison. Furthermore, traditional medical technologies do not provide physicians with a proposed course of action to “solve” the medical problem at hand, they only supply additional information on the patient’s condition, which can be interpreted in a pretty straightforward manner by trained professionals. Since DDSSs often go “a step further” by suggesting possible diagnoses, understanding the results they produce is certainly a much more complex endeavor.

Another problem in the relationship between humans and machines, which descends from the opaque nature of ML, is that it may hinder the correct conveyance of information to the patient, and consequently impede accounting for the patient’s values and preferences. This issue is not novel to the medical field. The difficulties in guaranteeing an “informed choice” have long been discussed by scholars (16). Conveying medical information to patients can often prove challenging due to the asymmetry in technical knowledge. Furthermore, the physicians must also take into account the patient’s opinions when deciding the correct course of action. This reciprocity in the information exchange, which is at the basis of the shared decision-making process between patient and physician, creates significant complexity, and it lacks a clear uniform solution. From this point of view, the AI’s lack of explainability does not create an entirely “new” problem, but it does further aggravate the issue as the physician will struggle with understanding the meaning of the machine’s output and thus with the two-way information exchange.

## 4 Why explainability should or should not be pursued

It is important to question whether explainability is a truly significant objective from a practical viewpoint, and if so, to what extent, i.e., how explainability should be balanced with other interests in case of conflict. Explainability, in fact, sometimes requires a trade-off, e.g., with accuracy (17).

A study by Nagendran et al. (18) aimed at assessing how additional information influences doctors’ prescriptions, found that while AI is extremely influential, explainable AI (XAI) is not more influential than AI by itself. It was also found that there was no correlation between self-reports of the influence of XAI and actual influence. While not many studies of this kind have been conducted, it is important to reflect on whether an explanation would concretely be beneficial from the physician’s standpoint or whether it only appears to be needed because of preconceived notions.

It must be highlighted that, even if XAI did not have significant influence over physicians, it would still be necessary for the correct implementation of evidence-based medicine^4^ (EBM), which is considered to be the “golden standard” of medicine (19). “Informed choice”, to be considered valid, should always be based on EBM (16). Without explainability, in fact, one could not verify whether the decision was taken based on a correct interpretation of relevant guidelines and literature. It is crucial to keep in mind that in many legal systems (both in the US (20) and in the EU (21)) EBM plays a significant role in the assessment of medical malpractice. Being able to check what scientific studies were specifically recalled to produce a certain output, could reassure physicians that their decision abides by the “golden standard”, and thus that they are unlikely to face liability. In this sense, explainability could still impact physicians’ trust and reduce defensive medicine practices, consequently favoring more widespread AI uptake and overall better results for the healthcare system.

When it comes to patients it is, instead, undoubtable that understandable information has intrinsic value since it is crucial in protecting patient autonomy. In this sense, explainability certainly acquires relevance, and it cannot be disregarded. The physicians will have to continue to intermediate between medical knowledge and patient, as they always have done, even when such knowledge is the result of automated processing, and XAI can ease this process. Once again, it should be pointed out that whenever “informed choice” is found to be lacking, the physician could be liable. Shared decision-making is linked with greater compliance with the treatment plan (22), as well as overall better health outcomes and levels of satisfaction (23). Predictably, it has long been demonstrated that inadequate physician-patient communication is among the primary reasons for lawsuits against physicians when an adverse outcome occurs (24, 25). It must also be remarked that defensive medicine goes hand in hand with perceived exposure to patient complaints (26, 27). Since implementing informed choice, through better physician-patient communication, can potentially reduce medical malpractice lawsuits, it may also discourage physicians from engaging in defensive medicine practices. In light of all of this, guaranteeing patient autonomy not only serves an ethical purpose but can also influence physician behavior.

For the very sake of patients, though, it must also be questioned whether in certain cases they would prefer to sacrifice explainability in the name of better performance. This choice might theoretically be left for them to make e.g., by asking the patient to choose between a highly-accurate yet unexplainable system and a more understandable yet less successful one. It is questionable whether presenting this choice and informing the patient of the benefits and risks of each option could, in itself, fulfill the requirements of “informed choice”. Insofar as AI healthcare systems are not validated by the scientific community, in such a way that they themselves constitute evidence-based medicine, this solution seems insufficient.

## 5 The EU legal framework

The uncertainty surrounding explainability is of particular note considering that it is debated whether it should be considered a legal requirement or not. Explainability itself is rarely mentioned by the EU legislator, as most of the focus tends to be placed on transparency instead.

Art. 22 of the General Data Protection Regulation (GDPR) (28), which regulates automated decision-making, does not impose transparency. Recital (71) does mention a “right to an explanation”, but it is not mandatory, and could be more easily interpreted as a mere recommendation to the developers of AI to provide explanatory information (29). Art. 22.3 does demand, in the exceptions mentioned by Art. 22.2.a and 22.2.c, suitable measures to guarantee a right to human intervention, to express his or her point of view, and to contest a decision, all of which fall partly in line with the need for explainability. Articles 13 and 14 of the GDPR also impose informational duties, namely about the existence of automated decision-making, meaningful information about the logic involved, and the significance and envisaged consequences of the data processing. It is unclear what such information should consist of.

The recently approved AI Act affirms the principle of human oversight for high-risk systems (Art. 14), which to be truly enforced would require a certain degree of explainability. It also provides a duty of transparency that is tailored based on the risk level (13). For high-risk systems, for example, Art. 13 states that sufficient transparency shall be provided to those using the system under their authority. It is interesting to note that Art. 13 prescribes that instructions for use should contain information about “the technical capabilities and characteristics of the high-risk AI system” so as to “provide information that is relevant to explain its output.”

Moreover, Art. 86 of the AI Act (Right to explanation of individual decision-making), provides affected persons subject to a decision which is taken by the deployer on the basis of the output from a high-risk AI system, the right to obtain from the deployer clear and meaningful explanations of the role of the AI system in the decision-making procedure. However, such provisions provide a right to an explanation only of the role of the AI, not of the functioning or the output produced by the system. Furthermore, it concerns only a limited list of high-risk systems, which does not include most of the AI systems used in healthcare, and in particular, does not include medical devices having an AI component.

The legislator has failed to provide specific requirements concerning explainability. This is largely due to the precise fact that explainability is still of somewhat debatable usefulness and, more importantly, that there is no consensus on what it should consist of, as the type of required explanation might change depending on both the context and the envisioned recipient.

## 6 Potential XAI solutions

Attempts have been made to build “white box” or “glass box” models which, as the names suggest, aim at transparency in the reasoning process of the machine. Approaches have been proposed both by computer scientists and social scientists, the latter are especially aimed at lay people who do not have specific technical knowledge of AI systems.

A classic distinction that can be drawn is between intrinsic and *post hoc* explanation. In intrinsic explanations, it is the model itself that is structured in a way that makes it understandable. Post hoc explanations are instead obtained through external methods when analyzing the model (30).

Another possible classification is that between feature-based, textual, and example-based explanations (30). What could be a particularly interesting explanation for physicians would be a contrastive explanation, which pinpoints the features that have most influenced the outcomes and what values would have led to different results (6). This might, in fact, help reconstruct what would have been the logical process of a human, thus making it easier for the physician to decide whether he agrees or not with such reasoning.

A particular type of explanation that can be recalled is also that of the heat map. This post-hoc explanation that is used in medical imaging can show the areas of the image that the AI has concentrated on in determining its conclusion (31). This may help physicians who, through the analysis of said maps, could understand what the machine has reasoned on, allowing for some understanding of the algorithm. At the same time, some authors have pointed out that it is actually quite difficult to interpret these maps, and that humans tend to pick and choose among the information they’re shown, to confirm their preconceived hypothesis (32).

A problem that almost all explanation models share is that they are not properly tested for their effectiveness and that they usually leave a gap in the interpretation process, that is left to the human’s intuition to fill (e.g., bridging the gap between what the machine looked at, and why it did) (31). Though subject-centric, tailored explanations, focused on singular outcomes (local explanations) would be the most beneficial option for stakeholders, it seems that explanations are currently better suited for verifying the overall functioning of the machine (global explanations), e.g., determining whether the AI is focusing on relevant data or meta-data (31).

Finally, an additional problem that has been highlighted by the literature (among them, Nazir et al.) (33) is the so-called trade-off between model accuracy and model interpretability that usually characterizes ML approaches. Despite the fact that a lack of interpretability does not necessarily prevent explainability, it may still make it less complete or effective (34).

## 7 Conclusion

The lack of explainability of AI systems further complicates pre-existing issues in the human-machine relationship that have long been discussed in the healthcare field. Though it seems that, at times, the relevancy of these issues is somewhat over-estimated, it is undeniable that solving them would be beneficial in clinical care. Effective XAI would help in pursuing evidence-based medicine solutions and productive information exchanges between physician and patient, which are both fundamental features of the “informed choice” approach. These elements are not only relevant in themselves, but they also participate in shaping the behaviors of physicians, due to the possible consequences in terms of liability, and thus have an immediate and direct effect on the healthcare system as a whole.

XAI encompasses a variety of approaches, all of which seem so far to be somewhat unsatisfactory. The focus, when it comes to the healthcare sector, should be on allowing physicians to reconstruct what factors were crucial in reaching the output, so as to allow them to better evaluate the conclusion itself.

## Data Availability

The original contributions presented in this study are included in this article/supplementary material, further inquiries can be directed to the corresponding author.

## Footnotes

1 Machine learning is a subcategory of AI and it encompasses multiple different approaches, all of which entail the ability of the machine to learn autonomously (6).

2 Leslie at al. state that Decision-Automation Bias occurs when “implementers defer to the perceived infallibility of the system and thereby become unable to detect problems emerging from its use for reason of a failure to hold the results against available information.” In Automation-Distrust Bias, instead, “users of an automated decision-support system may tend to disregard its salient contributions to evidence-based reasoning either as a result of their distrust or skepticism about AI technologies in general or as a result of their overprioritisation of the importance of prudence, common sense, and human expertise” (14).

3 Unpredictability can be defined as the “inability to precisely and consistently predict what specific actions an intelligent system will take to achieve its objectives, even if we know [the] terminal goals of the system” (35).

4 The idea behind EBM is that that the best results can be obtained from a combination of evidence hierarchy and clinical practice guidelines, thus placing great weight and value on medical literature (36).

## References

[1] CB Insights Research. *Big Tech in Healthcare: How Amazon, Google, Microsoft, & Nvidia are Looking to Transform drug R&D, Primary Care, and More.* (2024). Available online at: https://www.cbinsights.com/research/report/big-tech-healthcare-amazon-google-microsoft-nvidia/ (accessed December 7, 2024).

[2] Zikos D DeLellis N. CDSS-RM: A clinical decision support system reference model. *BMC Med Res Methodol.* (2018) 18:137. 10.1186/s12874-018-0587-6 30445910 PMC6240189

[3] Silva S Hak F Machado J. Rule-based clinical decision support system using the OpenEHR standard. *Proc Comput Sci.* (2022) 201:726–31. 10.1016/j.procs.2022.03.098

[4] Yu K Beam A Kohane I. Artificial intelligence in healthcare. *Nat Biomed Eng.* (2018) 2:719–31. 10.1038/s41551-018-0305-z 31015651

[5] Pasquale F. *The Black Box Society: The Secret Algorithms That Control Money and Information.* Cambridge: Harvard University Press (2015).

[6] European Parliament, Lagioia F Sartor G. The impact of the general data protection regulation on artificial intelligence. *Publ Office.* (2020): 10.2861/293

[7] European Commission. *Directorate-General for Communications Networks, Content and Technology, Ethics Guidelines for Trustworthy AI.* Brussels: European Commission (2019). 10.2759/346720

[8] Floridi L Cowls J Beltrametti M Chatila R Chazerand P Dignum V AI4People-An ethical framework for a good AI society: Opportunities, risks, principles, and recommendations. *Minds Mach (Dordr).* (2018) 28:689–707. 10.1007/s11023-018-9482-5 30930541 PMC6404626

[9] Barredo Arrieta A Díaz-Rodríguez N Del Ser J Bennetot A Tabik S Barbado A Explainable artificial intelligence (XAI): Concepts, taxonomies, opportunities and challenges toward responsible AI. *Information Fusion.* (2020) 58:82–115. 10.1016/j.inffus.2019.12.012

[10] Esposito E. Does explainability require transparency? *Sociologica.* (2022) 16:17–27. 10.6092/issn.1971-8853/15804

[11] Markus A Kors J Rijnbeek P. The role of explainability in creating trustworthy artificial intelligence for health care: A comprehensive survey of the terminology, design choices, and evaluation strategies. *J Biomed Informatics.* (2021) 113:103655. 10.1016/j.jbi.2020.103655 33309898

[12] Trist E Bamforth K. Some social and psychological consequences of the longwall method of coal-getting: An examination of the psychological situation and defences of a work group in relation to the social structure and technological content of the work system. *Hum Relat.* (1951) 4:3–38. 10.1177/001872675100400101

[13] European Union. Regulation (EU) 2024/1689 of the European Parliament and of the Council of 13 June 2024 Laying Down Harmonised Rules on Artificial Intelligence and Amending Regulations (EC) No 300/2008, (EU) No 167/2013, (EU) No 168/2013, (EU) 2018/858, (EU) 2018/1139 and (EU) 2019/2144 and Directives 2014/90/EU, (EU) 2016/797 and (EU) 2020/1828 (Artificial Intelligence Act). Maastricht: EU (2024).

[14] Leslie D Rincón C Perini A Jayadeva S Borda A Bennett S *AI Fairness in Practice.* London: The Alan Turing Institute (2023).

[15] Contissa G. Automation and liability: An analysis in the context of socio-technical systems. *i-lex.* (2017) 11:17–45.

[16] Marteau T Dormandy E Michie SA. measure of informed choice. *Health Expect.* (2001) 4:99–108. 10.1046/j.1369-6513.2001.00140.x 11359540 PMC5060053

[17] Petkovic D. It is not “accuracy vs. explainability”—We need both for trustworthy AI systems. *IEEE Trans Technol Soc.* (2023) 4:46–53. 10.1109/TTS.2023.3239921

[18] Nagendran M Festor P Komorowski M Gordon A Faisal A. Quantifying the impact of AI recommendations with explanations on prescription decision making. *npj Digit Med.* (2023) 6:1–7. 10.1038/s41746-023-00955-z 37935953 PMC10630476

[19] Sackett D Rosenberg W Gray J Haynes R Richardson W. Evidence based medicine: what it is and what it isn’t. *BMJ.* (1996) 312:71–2. 10.1136/bmj.312.7023.71 8555924 PMC2349778

[20] Mackey T Liang B. The role of practice guidelines in medical malpractice litigation. *Virtual Mentor.* (2011) 13:36–41. 10.1001/virtualmentor.2011.13.1.hlaw1-1101 23121815

[21] Zerbo S Malta G Argo A. Guidelines and current assessment of health care responsibility in Italy. *Risk Manag Healthc Policy.* (2020) 13:183–9. 10.2147/RMHP.S238353 32210649 PMC7073368

[22] Sandman L Munthe C. Shared decision making, paternalism and patient choice. *Health Care Anal.* (2010) 18:60–84. 10.1007/s10728-008-0108-6 19184444

[23] Hughes T Merath K Chen Q Sun S Palmer E Idrees J Association of shared decision-making on patient-reported health outcomes and healthcare utilization. *Am J Surg.* (2018) 216:7–12. 10.1016/j.amjsurg.2018.01.011 29395026

[24] Levinson W Roter D Mullooly J Dull V Frankel R. Physician-patient communication. The relationship with malpractice claims among primary care physicians and surgeons. *JAMA.* (1997) 277:553–9. 10.1001/jama.277.7.553 9032162

[25] Lindor R Kunneman M Hanzel M Schuur J Montori V Sadosty A. Liability and informed consent in the context of shared decision making. *Acad Emerg Med.* (2016) 23:1428–33. 10.1111/acem.13078 27607573

[26] Goetz K Oldenburg D Strobel C Steinhäuser J. The influence of fears of perceived legal consequences on general practitioners’ practice in relation to defensive medicine - a cross-sectional survey in Germany. *BMC Primary Care.* (2024) 25:23. 10.1186/s12875-024-02267-x 38216861 PMC10785451

[27] Frakes M Gruber J. Defensive medicine: Evidence from military immunity. *Am Econ J. Econ Policy.* (2019) 11:197–231. 10.1257/pol.20180167 31632594 PMC6800742

[28] European Union. *Regulation (EU) 2016/679 of the European Parliament and of the Council of 27 April 2016 on the Protection of Natural Persons with Regard to the Processing of Personal Data and on the Free Movement of Such Data, and Repealing Directive 95/46/EC (General Data Protection Regulation).* Maastricht: EU

[29] Wachter S Mittelstadt B Floridi L. Why a Right to explanation of automated decision-making does not exist in the general data protection regulation. *Int Data Privacy Law.* (2017) 7:76–99. 10.1093/idpl/ipx005

[30] Chaddad A Peng J Xu J Bouridane A. Survey of explainable AI techniques in healthcare. *Sensors (Basel).* (2023) 23:634. 10.3390/s23020634 36679430 PMC9862413

[31] Ghassemi M Oakden-Rayner L Beam A. The false hope of current approaches to explainable artificial intelligence in health care. *Lancet Digit Health.* (2021) 3:e745–50. 10.1016/S2589-7500(21)00208-9 34711379

[32] Bornstein A. *Is Artificial Intelligence Permanently Inscrutable? Nautilus.* (2016). Available online at: https://nautil.us/is-artificial-intelligence-permanently-inscrutable-236088/ (accessed December 6, 2024).

[33] Nazir S Dickson D Akram M. Survey of explainable artificial intelligence techniques for biomedical imaging with deep neural networks. *Comput Biol Med.* (2023) 156:106668. 10.1016/j.compbiomed.2023.106668 36863192

[34] Rudin C. Stop explaining black box machine learning models for high stakes decisions and use interpretable models instead. *Nat Mach Intell.* (2019) 1:206–15. 10.1038/s42256-019-0048-x 35603010 PMC9122117

[35] Yampolskiy R. Unpredictability of AI: On the impossibility of accurately predicting all actions of a smarter agent. *J AI Consci.* (2020) 07:109–18. 10.1142/S2705078520500034

[36] Knaapen L. Evidence-based medicine or cookbook medicine? Addressing concerns over the standardization of care. *Sociol. Compass.* (2014) 8:823–36. 10.1111/soc4.12184

